# American Dental Association

**Published:** 1882-09

**Authors:** E. G. Betty


### AMERICAN DENTAL ASSOCIATION.

### Twenty-second Annual Meeting, at Cincinnati. Ohio, August 1st, 2d, 3d, and 4th, 1882.

### REPORT OF DISCUSSION BY E. G. BETTY, D.D.S.

The morning of the first day was consumed in organization of the meeting, miscellaneous business, etc.

In the afternoon, Section 3 reported a paper on "Nomenclature and Terminology," by Dr. Wm. H. Atkinson, of New York, the gentleman reading his paper. Considerable discussion ensued, not upon the paper, but upon the propriety of referring it to a special committee of five, to pass upon its merits and find out if possible, anything in it that would entitle it to a place in the schedule of dental subjects. It was finally ordered to follow preceeding reports upon the same subject and be published in the transactions.

### SECOND DAY—MORNING SESSION.

On motion of Dr. Atkinson, the association accorded the privileges of the floor to Prof. Mayr, of Springfield, Mass., scientific editor of the *New England Journal of Dental Science*, and a thorough chemist.

PROF. MAYR stated among other things that he had for several years past studied chemistry in relation to dental caries. He has found that in the black, decayed mass of caries, there still remained sixty per cent. of the lime salts. This proved to him that all theories about the total solution of the lime salts in decay are erroneous.

Section 4, through Chairman E. T. Darby, reported a paper on "Some Thoughts and Suggestions on the Irregularities of the Teeth," by Dr. W. M. Morrison, of St. Louis. The following are a few of the points of this excellent paper.

It is an exceedingly stupid thing to extract the six-year molars. It is a rare occurrence to see a full set of good teeth with a correct articulation. It is absurd and ridiculous to suppose that the continued extraction of a certain tooth will not produce hereditary tendencies and effects in the future. The public is clamorous for the destruction of the mouth by extraction of the natural teeth and the substitution of artificial, and it is a disgrace to the profession that so many dentists accede to the demand. Many have become enthusiastic over Madam Patti's small mouth and beautiful teeth, whereas, in reality, her mouth is a deformity. The teeth are irregular, one canine being way out of the arch and it is difficult for her to get the teeth together. Irregular teeth are very easily moved, if the force is applied in the right direction. Annular bands made of thin strips of platinum are cemented to the teeth with oxyphosphate of zinc and are to remain until operation is complete. The band is the base of operations, the free end of the attached screw operating against the tooth to be moved. There is no better force than the old fashioned screw, lever and wedge. All we want is a correct application of the force they supply. The bands of platinum can be made thin enough to pass between the teeth, thus obviating the necessity of cutting the natural tooth. Several levers and jackscrews can be used in the mouth at the same time. Regulating should be done by the expansion of the arch and seldom by its contraction. After the teeth have been moved into their proper positions, retaining bands should be made as described above.

Dr. Atkinson. Am sorry to see so little disposition of the members for many to be on the floor at once.

The aphorisms in Dr. Morrison's paper are orthodox and are correct with but few exceptions. I do not agree with him in the idea that the jackscrew should be used. It is true that the force applied should be in the direct line of the tooth. A slight force continually acting will move the most refractory set of teeth. Ten days to two weeks is enough time in which to move the teeth their own breadth, in the mouth of a patient thirty years of age. The constant irritation incident upon moving the teeth is the sole cause of making them sore. In undertaking the regulation of a set of irregular teeth, we must study what we do, and guess at nothing. We can now accomplish in days what we formerly consumed weeks in doing. (The doctor showed specimens of regulating plates made of Reese cast gold alloy, dental base, pamphlet and circulars describing the method of taking impressions for this base.) The wax plate is carried over the molar teeth on the model and enough wax built upon it to get articulation with opposing teeth. From this the regulating plate is made. It is essential to get complete control of the patient and not let him go home with levers and watch-keys, to do his own regulating. A band of platinum is run round outside of the teeth and attached to plate at the molars on either side. Holes and slots are cut in this band, from which the force is applied. By this method the teeth can be regulated in a short time.

Dr. Pierce. This method is very fine and will do for some teeth. In other cases it can have no applicability at all. We cannot enforce the same rule in all instances. The jackscrew will sometimes turn a tooth with but little trouble and pain; at other times the elastic bands are preferable.

Dr. Shepard. Dr. Atkinson stated in his remarks that the regulating plate should remain in the mouth until the work is done. I do not agree with him in that. The plate must be removed at intervals for cleanliness' sake. Any appliance that can not be removed by the patient is not a good one, for cleanliness is an absolute necessity. Plates can be made that will perform the work required and at the same time offer every facility for removal and cleaning. The retention of a plate in the mouth is dangerous to the teeth, the surrounding tissues and to the health of the patient.

Dr. Atkinson. The patient can be clean without the removal of the plate, and I know my patients are as clean as those of any other dentist.

Dr. McKellops. This is a subject in which I take a great deal of interest. I have the simplest method in use and it further admits of ready removal by the patient himself for cleansing. I got the method at the International Congress in London, in 1881. I refer to Dr. Coffin's split plate. I saw in Dr. Coffin's office models of 2,500 cases that had all been successfully treated upon this system. These as demonstrating the method were exhibited to the profession and received a hearty indorsement. The whole thing rests upon a small piece of piano wire.

DR. W. H. MORGAN. What is the guage of that wire?

DR. BARRETT. In an old piano the wires are thinner than those now used in pianos.

VOICE. Guage fifteen (15).

DR. MCKELLOPS. The system is somewhat on Dr. Atkinson's plan. To begin with the impression must be accurate. The cup is fitted to the mouth and then filled with English gutta-percha softened in hot water, plunged in cold water to chill the surface. It is then introduced into the mouth and the patient instructed to bite it into place. After removal the impression is again immersed in cold water to contract the gutta-percha to its normal bulk. A plaster model and wax plate are then made. The steel wire is bent in spring form something like a double letter S and to fit inside the arch. The ends are flattened, coated with tin and immersed in the wax plate. The whole is then flasked, vulcanized and adapted to the mouth. The plate is then divided with a saw, down the median line, replaced in the mouth to remain two or three hours, when the spring is spread a little. In a child's mouth one night is sufficient to spread a crowded arch.

It is possible, by this means, to regulate the teeth of a patient forty years of age. The spreading of the arch separates the teeth better than any other way, and ligatures can be applied to change the position of any tooth, or teeth, desired. The regulation is very easily done, and occasions the least amount of pain and soreness. A child can remove and replace the appliance without assistance, and clean it. There is no telling what can be accomplished by Dr. Coffin's method. If, as has been stated, this system has been in use in Boston for the last ten years, I have not seen it there. I first heard of it, and saw it demonstrated in London. Another apparatus I would like to present was described in a paper by Dr. Patrick at the Illinois State Dental Association. It consists of a band of gold running outside and in front of the arch, steadied at the bicuspids by little rings between the teeth. The bar is made to slide forward through slots in the side pieces, and can be fixed by little thumb-screws pressing against the bar. The teeth to be moved are hitched to the bar, it moving as desired, carrying the teeth with it.

Dr. Noel mentioned an apparatus in use by Dr. C. R. Taft. The plate is of rubber, covering the grinding surface of back teeth, and occluding with opposing teeth, thus giving room for the teeth to be moved to swing clear.

Dr. McKellops. Dr. Coffin's article was published in the Dental Register not long ago, and I advise every member to study it thoroughly. I would like right here to say a word for English Dentists. When at the Congress last summer, I found them to be clever, courteous and generous. Nothing was too good for them to do for the visiting Americans The English physicians, also, treated the Americans in the most generous manner. Such men are Turner, Rodgers, Tomes, Oakley, Coles, and many others whose names I cannot at present recall. It was come here and go there, and feast from morning till night.

Dr. Hurd. This is a subject in which I am much interested, and am sometimes successful, sometimes not.

Dr. Shepard. I rise to indorse the Coffin method. Dr. D. M. Clapp was at one time with Dr. Coffin, and brought his method to Boston ten years ago. I exhibited the method two years ago, and have used it more or less extensively ever since But not until last summer, in London, did I appreciate its full capabilities. The credit of it belongs to Dr. Coffin. J. D. White's split plate is in "Kingsley's Oral Deformities," but is so different that it cannot be mentioned in the same connection. I wish, also, to add some points not touched upon by Dr. McKellops. I use three sizes of wire, instead of one, and get that many degrees of pressure. Am also in the habit of coating the whole spring with tin, to avoid oxidation. The steel wire becomes blackened with a smooth oxidation unlike the roughness of an iron wire. It is necessary to make every bend in the spring a curved line, and that nicking and bruising the wire is to be avoided. The practical points in this method can only be had by demonstrating, and I hope Dr. McKellops will have the time to do it. I regard the method as the most valuable in use.

Dr. Field. I am quite surprised that Coffin's plates are not more extensively used by my colleagues. They have been used in Detroit for some time past. I have been truly and well repaid by my trip to London. I was taught, when a boy, that the sun rises in Boston. I am surprised that Boston has not let us have the benefit of this method that has been in use there for ten years past. I hope it will profit by London's example.

Dr. Darby. In covering the molars with the regulating plate, it is essential to cap them all, because an uncapped molar will elongate and produce mal-occlusion. Dr. Atkinson forgot to mention that point. In one case one or two of the molars and bicuspids were not capped, and in six months they elongated one-eighth of an inch. I thickened the plate, and in another six months the elongation amounted to one quarter of an inch. In making my plates I cover all the back teeth, and allow the band to run around in front of the arch, just touching the labial surfaces of the teeth, and under the lip. Once a tooth is moved it should be held firmly in place, and not allowed to move back and forth.

Dr. Eames, In Dr. Patrick's method, as described by Dr. McKellops, there is simply a band with loops between the teeth to get a fixed point from which to work. The band moves back and forth through slots in the loops and is set by thumb-screws.

Dr. Butler. In the methods described by Dr. McKellops, the one point overlooked was the presentation of a practical case. I have tried all kinds of appliances, and am sorry that Dr. McKellops has failed to give a single practical illustration of the Coffin system. The Farrer method is all very nice on paper, but practically it is good for nothing, except to a limited degree. One method will not meet the requirements of all cases, and Dr. Atkinson's cast-iron rule will not make all cases come out perfectly.

Dr. Coffin's method has the merit of being at once the simplest and least expensive. No bungler will make any method work successfully, for regulation of the teeth requires considerable skill. Coffin's plan covers the most ground, and can be made by those with little experience. It also allows removal for cleaning. The principle advantage it posesses is that those teeth not to be moved can remain as they are. The Patrick band system moves teeth that should not be touched. I agree with Dr. Darby that once a tooth is moved it should be held firmly in place. It is far more difficult to retain the teeth in position than it is to move them in the first place. If the whole session was consumed in the discussion of this subject, the time would be well spent.

Dr. Eames. I regard Dr. Patrick's system as the simplest and best, because the impression and band can be made in one sitting, and in an hour or so. A knowledge of the lever will at once explain this method of regulating teeth.

Dr. Dorrance. I wish to caution those using the Coffin system not to employ too large a wire. A large wire exerts too much force, causing a great deal of irritation and pain, and is, furthermore, apt to do damage. A small wire will answer every purpose. It is essential to get a bearing upon all the teeth. Any tooth may be moved in or out or rotated by the simple spring, and without interfering with the plate or its removal by the patient. With silk ligatures attached to a hook or wire, as the case may be, a tooth can be moved with ease, and without trouble.

Dr. McKellops. I said before that the plate is to be worn a few hours before it is split. If the Coffin method is carefully and skillfully used, there can be no danger of causing either damage, pain, or irritation. Each system possesses more or less advantage, but my preference is for the Coffin system.

Dr. G. W. Keely. In my bungling way I have done a little regulating during the last quarter of a century. Some twenty years ago I made a plate for a young miss of nine years of age; the superior incisors were locked behind the inferior incisors. I made the plate to cover the grinding surface of the molars, and carried a band around the front. The incisors were hitched to the band with ligatures, introducing the plate at 4 o'clock P.M. The next morning the teeth had reached their proper place, and the work was done. At the present time I would proceed somewhat differently. I would make the plate thicker on the palate, cut slots and fit into them pieces of sea-tangle tent. This material, when saturated with moisture, swells considerably, and in a confined space is capable of exerting considerable pressure.

Dr. Pierce. In the regulation of teeth, economy of time and simplicity of method are very desirable. I can operate very satisfactorily by using narrow strips of platinized gold. Drill a small hole at the margin of the gum on the first molar, and another on the incisor to be moved. Sharpen the ends of the platinum strips and spring it into the holes. The elasticity of the bar will give force enough to move the tooth. The strip is not at all in the way, because it lays along the arch and does not interfere with the tongue.

Dr. G. W. Keely. In many cases it is necessary to remove the first molars; the second will come forward and fill their places, thus giving room for the third molars, whose life is usually crushed out for want of space.

Dr. Crouse. I have nothing new to offer in the regulation of teeth, and usually do the best I can under the circumstances. In one case I had, the arch was very crowded and peaked, the lower teeth so close together laterally that there was not room for the tongue. The upper jaw I spread a distance of one and one-quarter inches; the lower, one and one-half inches.

Dr. Markline. Judging from the appliances that have been exhibited, I imagine they are all intended for the expansion of the arch. I should very much like to know if they can also be used for contracting the arch. It is very much easier to move teeth forward, but very difficult to move them backward.

Dr. McKellops. There is a great variety of ways of applying the Coffin method. It can also be used to contract the arch, if the operator knows how to apply the principle in all its variety.

Dr. Markline. In one case I had to deal with, the only anchorage was the superior second molars, while ten teeth in front of them had to be moved backwards. I would like to know if it is possible to move those ten teeth with only the second molars as a base.

Dr. Darby. I should first move back the first molars, and then the bicuspids, one after another, by means of wedges, holding each tooth in place as it was moved.

Dr. Morrison. In 1865 Dr. McKellops brought the original Coffin plate from Europe; the plate was made to cover the molars as now. I have used them ever since, and have accumulated a great many in my laboratory. These plates, and others that are now made, are awkward and bunglesome, and, moreover, are apt to slip out of position, especially when the crowns of the teeth are short. The system I have described in my paper does away with all that.

SECOND DAY—AFTERNOON SESSION.

Section six being called for, two papers were reported.

Dr. A. O. Rawls' paper on Pulpless Teeth. In this class of teeth there are two tissues more or less intact and devoid of life, the enamel and dentine. The one presents but few breaks in the nutrient capability, the other many. Whether an abscess exists or not, there is a break in the continuity of the nutritive process. An abscess with a dead pulp as the cause, exhibits a dentigerous cyst or abscess sac, which may be situated almost anywhere on the root. (As Dr. Rawls' paper will be found in the transactions for 1882, the reader is referred to them. Rep.)

Dr. Harlan then read his paper, which may be considered as a continuation of the above, being, however, but one phase of it.

Dr. Coffin, of London, has stated that when there is no fistulous opening, the sac is to be injected with hydrogen di-oxide. This agent is prepared as follows: Add one part of chemically pure sulphuric acid to five parts of distilled water; gradually add to this moist, purified barium di-oxide until there is only a slight acid reaction. Barium sulphate is precipitated; the remaining solution is then filtered. There are two ways of concentrating this solution of the di-oxide. First, is to freeze the solution and remove the frozen portion; the remainder is the concentration. Second, place the solution in a vacuum and evaporate it over sulphuric acid. The remaining part not evaporated is the di-oxide in solution. It must be kept in a cool, dark place. Should the sac be filled with pus, inject the hydric oxide. The oxygen which is evolved rapidly distends the sac and forces out the pus. The volatile oil of eucalyptus is then introduced with a shred of cotton. Proceed as usual afterwards.

Prof. Mayr. Dr. Rawls said in his paper, "are we not presuming too much in the use of such terms as 'vis vitæ,' 'physico-chemical,' etc.?" I should like very much to have a definition of those terms.

Dr. Rawls. This vis vitæ is a mysterious and intangible something, that we think of and express as vital force.

Prof. Mayr. I think it is an empty word.

Dr. Rawls. So do I.

Prof. Mayr. It is worse than useless to use words and terms that mean nothing. About hydrogen di-oxide: I suggested at the Connecticut State Dental Association that it could be used to bleach teeth. There was such a difference of opinion expressed that nothing came of the matter. I said that it could be put into the cavity, but found that the rapid evolution of oxygen would burst the tooth. In the enamel of the tooth there is 97 per cent. of lime salts, one to two of animal matter, the balance water, so that osmosis will take place but very slowly. Now, what discolors the tooth? That, also, is a mooted question. I imagine that hematine under some form is the cause. There are no red corpuscles in the tooth, but there circulates lymph and serum, and these contain all the elements of the red corpuscles. The anti-septic properties of the hydrogen di-oxide is not due to oxygen, but to ozone. If we could get ozonized water or air, it would have the same effect as the hydric per-oxide which is so difficult of preparation.

Dr. Ingersoll. The remarks I have to make have already been offered to the profession in a series of papers in the dental journals.

1. Alveolar abscess distinct from alveolar ulceration. 2. Alveolar ulceration distinct from alveolar abscess. (A third form the reporter could not catch, which the doctor will surely tell anyone asking. Rep.) The question as to these differences arose from the fact of so many failures in the treatment of alveolar abscess. I wish especially to call attention to the ulceration beginning at the apex of the root, and proceeding to the margin of the process. No pulp dies without a cause. The form of disease which follows is various, it may be a true abscess, it may be an ulceration without suppuration, or may be a salivary calculous.

(Possibly the speaker meant sanguinary calculous. Rep.) In one case, I had treated for alveolar abscess for some time, and failed. I extracted the tooth and found upon the root a dark, hard substance. What was it? It could not be a salivary calculous, and could not be caused by gravity, for the tooth was above. In analyzing I found some things not found in salivary calculous, namely: coloring matter. This coloring matter is derived from the hematine of the blood; hence the name *sanguinary* calculus. Sanguinary calculus is never found in an alveolar abscess, but always in an alveolar ulceration. There are instances in which the sanguinary and salivary calculi touch each other on the same root; they do not unite, but remain separate with a distinct line of demarkation. The sanguinary calculus can be known by its very dark color and extreme hardness.

Voice. Is it not possible that the sanguinary calculus may derive some, or part of the constituents from the breaking down of the alveolus?

Dr. Ingersoll. It is possible that the calculus may derive part of its substance from that source, but the amount of the calculus is greater than the alveolus could supply.

Dr. Pierce. When these hard deposits are found away down on the root, they cannot be derived from the saliva: the process is always found broken down, and sanguinary calculus is the proper name.

Dr. Fredericks. Dr. Ingersoll, how many of these cases have you examined?

Dr. Ingersoll. I have had several which I have analyzed, but have sent the specimens away for others to examine.

Dr. W. H. Morgan. Is not the presence of this calculus one of the characteristics of Riggs' disease?

Dr. Ingersoll. Yes.

Dr. Rehwinkle. It is one of the *most* characteristic symptoms.

Dr. Rawls. The idea that sanguinary calculus comes from the effusion of serum or fluid that contains the elements, as Dr. Ingersoll says, is true, just as such deposits are found in other parts of the body. In the kidneys, liver, lungs and brain, these deposits are found on the external parts of these organs, and not in their internal structure. They are, no doubt, the result of inflammation. Sanguinary calculus is not a *result* of Riggs' disease, but a concomitant. Tissues that are in a condition to be broken down may be broken down at a certain point, and the deposit will be found at that place. The tissue is incapable of reforming, because its nutrition is destroyed by a break in the continuity of the tissue. We are in the habit of teaching our students and patients that we can cure these cases and cause healthy tissue to reappear over dead tissues, or where there has been a break in the continuity of nutrition, regardless of dead substances over which they would necessarily have to grow.

Dr. Watt. Sanguinary calculus is not mysterious by any means. We have been stumbling over it for some time, and until Dr. Ingersoll gave it a name. It is found in all parts of the system. Dr. Samuel Martin, M.D., my old friend, and myself, once removed from the abdominal muscles, where it had been imbedded, a sanguinary calculus that weighed thirteen ounces. This, you will all admit, is something larger than is usually found upon the teeth. [Laughter.] Once a nucleus is formed, the calculus will increase in size right along. I confess that I do not know what causes the deposit of sanguinary calculus, but I imagine that it is due to inflammatory action, a death of part of the blood making it incapable of sustaining in solution all the mineral constituents. As long as the saliva contains free carbonic acid, it will hold in solution the lime salts. If the acid is removed the salts will deposit. The ordinary cause of salivary calculus is the presence of ammonia, and just as sure as it exists will there be a deposit. An alkaline wash, used to prevent tartar, takes up the free carbonic acid from the saliva (I speak with reference to mixed saliva) and the tartar still deposits.

Voice. What is the source of the ammonia?

Dr. Watt. The ammonia comes from a general breaking down of the tissues of the body. I sometimes think it is given off as an excretion from the salivary glands themselves. The breath also gives it off, the saliva dissolving it, thus acquiring the ammoniacal reaction.

Dr. Ingersoll. All the elements for the nutrition of the body are found in the blood. We find calcareous degeneration in various tissues of the body. Salivary calculus undoubtedly comes from the saliva, but the saliva itself is secreted from the blood.

Dr. Buckingham. It is not difficult to formulate a theory, but can we establish it upon a basis of facts? Wherever you have a dead nucleus, as a splinter of dead bone or a bullet, etc., imbedded in a tissue, there will be an accretion or deposition of the lime salts by a chemical or electrical process. The statement that when the liquor sanguinous circulating around the end of a root where there is inflammation and partially dead tissues gives off carbonic acid and deposits lime, is merely a theory. In the hydrogen di-oxide, spoken of by Dr. Harlan, there is a large portion of oxygen weakly combined with hydrogen. When decomposed the oxygen is given off in its nascent condition and is very active in its affinities. The ozone spoken of by Prof. Mayr, is two atoms of oxygen weakly combined so that a slight disturbance will set them at liberty. The ozone is usually represented as three parts of the molecule, with the fourth ready to be liberated. There is a theory that a tooth can be bleached by a mixture of sulphite of soda and borax ground together and put into the tooth after it has been cleaned. The theory is that the borax decomposes the sulphite of soda, liberating sulphurous acid which, having an affinity for oxygen, takes it from the tooth, in that way destroying the coloring matter. This was mentioned to me by Dr. E. C. Kirk, and I do not offer it as an indorsement, but merely to let others try it.

Dr. W. H. Morgan. Some one stated that the alveolar socket is not lined with periosteum, there being but one, and that on the root. I take issue with him, and declare there are two. There is no such thing as perfect success in treating alveolar abscess in dead teeth. I cannot understand that there exists a pus secreting surface, and cannot see that there can be such a thing as circulation of pus in the body. Pus is formed from elements in the blood after they have passed out of the blood at the seat of the disease.

Dr. Atkinson. I am very sorry to see that this body has degenerated into a crazy body—more crazy than Atkinson ever was. [Prolonged laughter.] We all think, but don't know how to present our thoughts. (The doctor proceeded at some length and being difficult to report, we give it up.)

Dr. Pierce. There are some local conditions that throw a little light upon the presence of sanguinary calculus. The tooth is abnormally yellow, the pulp cavity and the tubuli are filled up. Wherever there is this amorphous deposit there will be found pus, and sooner or later it makes its way to the surface.

### THIRD DAY—MORNING SESSION.

Section six being called, Dr. Odell read a paper on Diathesis and Cachexia.

The time will come when medical investigators must study the remote tendencies and peculiarities of the human family in order to discover the causes that produce disease. The future treatment in medicine will, and must be, prophylactic. This appears to be the idea in the paper. The discussion of Dr. Odell's paper, and those of the day before, was resumed.

Dr. Atkinson. There are two sides to the question of heredity. 1. The child derives its tendencies from its antecedents, modified by its surroundings. 2. That it acts from its own individuality, formed by its surroundings or environments. We must not be extreme, but take a middle ground in order to get at the base and form a rule by which we may successfully treat the matter. When we deal with remedies, they must have affinities making them capable of harmonizing with the requirements of the tissues through the process of digestion. In some respects there is so little difference between pathology and physiology that the one appears to be the other.

Dr. Fredericks. We do not know anything about how medicines act. Our knowledge of their action is impirical, and we only judge of their action by the effects they produce.

Dr. Atkinson. Food or medicines, in order to nutrify or cure, as the case may be, must have an affinity for the tissue to which they are applied. The assimilation of food and medicines is just in proportion to a harmonization of the affinities both of the tissues and the agents introduced.

DR. BUCKINGHAM. We use medicines just as oil is used on machinery; in order to assist nature in performing her functions. We do not know how medicines act, but when experiment gives us a uniform result, we set it down as a law that such and such a medicine produces a stated effect. Some medicines act as a whole, others by a chemical decomposition, a portion only being in action.

DR. WATT. These statements of Dr. Buckingham's are better than I could make. It will not do to say that we do not know how medicines act—the statement is too general. The idea of the action of medicines is a complicated one. We *do* know how *some* medicines act, and we *don't* know how to harness others. It will not do to merely say that medicine is medicine. I give, as an illustration, the treatment of malaria with arsenious acid. In growing Indian corn, the farmer acts by killing the weeds, if possible, to save the corn. So, in this malaria, there is a quantity of morbid tissue, and if we can kill this, on account of its lower vitality, we will save the healthy tissue. The arsenious acid kills the morbid tissue, the emunctories carrying off the the effete products and leaving the healthy tissues intact. Another instance, an anæmic girl, the anæmia due to one or more of several causes; there is a deficiency of the red blood corpuscles. We know that iron exists in the red corpuscles, and if we administer iron in some form or other, the red corpuscles will be developed. Even iron filings dusted over bread and butter, will produce the effect as well as any modified form of iron.

DR. BUCKINGHAM. How does the arsenic act, mechanically or chemically?

DR. WATT. It acts chemically by combining with the albumen.

DR. BUCKINGHAM. But how do you find this out?

DR. WATT. By analysis.

DR. FREDERICKS. When a physician assumes to treat a disease, he must know its cause. What is the cause of malarial fever?

Dr. Watt. Go to Dr. Sallsbury, and get the explanation from him.

Dr. Fredericks. Has the cause of malarial fever ever been proved?

Dr. Watt. It has been demonstrated.

Dr. Kulp. In taking medicines for the cure of a disease, it is necessary to take the form that will most easily assimilate. I would like to know if metallic iron eaten with bread and butter assimilates because it is taken as a food.

Dr. Odell. Dr. Watt made out a very clear case that he knows nothing about the action of medicines. Dr. Fredericks is right on his point, but when he asks the "how" it is too much for us to answer. Some medicines act by their mere presence. Sub nitrate of bismuth is hardly a medicine; it is a sort of slathering over of the intestinal canal. Arsenic has been proved to have an action upon the nervous system. Dr. Watt's explanation of the action of arsenious acid is not a true explanation.

Dr. Watt. I don't think that Dr. Odell heard what I said. We do know that these lower organisms die, and both the arsenic and effete tissue come out together. When the debris is cleared away in this manner, the nervous system has an opportunity to perform its functions. During a prolonged administration of arsenic, these metamorphic products are eliminated in larger quantities than under ordinary circumstances. We do not know anything in the sense that Dr. Odell criticises me, and I cannot subscribe to the doctrine that we know nothing about the action of medicines.

Dr. Odell. About all we know of the action of medicines is empirical. The science of the action of medicines is merely an arrangement of our empirical knowledge. We have learned the way some medicines act up to a certain point; we then experiment to learn how that action occurred. It is a difficult matter to establish the fact that a certain medicine acts upon a certain tissue and produces a given result. In the process of the oxidation of the blood we think we have the "how" of the action of medicines, and is the type upon which we base our theories.

Dr. Atkinson. We talk a great deal about tonics and stimulants. I wish to make a statement to show the different action of these upon the molecules of the body. The substance that impacts upon the molecules without combining with them, is a stimulant. The medicine that goes to the molecule and combines with it, and becomes a part of it, is a tonic. It is doubtful whether we can conceive of atoms ever having come into existence.

PROF. MAYR. In all our discussions we should adhere closely to facts. Dr. Watt says that the low organisms in the blood are killed by the administration of arsenic, and then eliminated. I had in my laboratory two bottles; one contained arsenious acid in solution, and was covered with mold; the other was filled with the arsenite of soda. I judge that the presence of the mold proves that arsenic does not kill low organisms.

VOICE. Were the bottles corked, or uncorked?

PROF. MAYR. They were uncorked.

DR. DARBY. Arsenious acid is said to act upon the pulp by catalysis. I used to think that it was absorbed. Dr. Flagg took $\frac{1}{25}$ of a grain of arsenic and devitalized the pulps of ten teeth in the mouths of ten different persons, using the one quantity of arsenic from one tooth to another. The remainder was placed upon the web of a frog's foot, and produced death. This demonstrated conclusively that the arsenic was not absorbed. The arsenic, at the conclusion of the experiment, was not weighed.

DR. BUCKINGHAM. I also know something about that frog. In testing for arsenic after the death of the frog Dr. Flagg and myself found arsenic in all parts of the animal. A very small quantity of arsenic produces a considerable effect. There is a chemical combination that takes place between the arsenic and the tissue, but it is weak and very easily breaks up, the arsenic attacking new tissue. I cannot say how it acts. It is true, however, that arsenic attacks the weak tissues first, as in a cancer. If it is applied to healthy tissue it is absorbed right through. From experiments only do we know that chemical combinations take place, but we know nothing about the chennical affinities making the combination possible. We only know matter by its properties and not by its substance.

Dr. Watt. Has not arsenious acid the property of co-agulating albuminous tissue?

Dr. Buckingham. Yes.

Dr. Watt. Would not the coagulation of the albumen make the absorption of arsenic through the pulp more difficult?

Dr. Buckingham. Yes.

Prof. Mayr. Has any gentleman ever observed arsenious acid coagulate albumen?

Dr. Buckingham. We know that tissue is killed by arsenious acid, for the arsenic is found in the inert mass. Arsenic will destroy the germ in seed; seed will not germinate that have been immersed in a solution of the acid. Arsenious acid appears to destroy vitality without chemical combination, the possibility of a removal of vitality in the tissue remaining; this is the case where but a small quantity of the acid is used.

Dr. Allport. So far as the action of medicines is concerned, there is a difference of explanation; they do act. We don't know why we live, we do, that's all. As I understand Dr. Flagg's experiment with the frog and pulps, the destruction of vitality was not from absorption or chemical action on the tissue of the pulp, but from irritation. In the pulps some weeks after a very slight quantity of arsenic was found and that, at the point of contact only. The irritation causes a flow of blood to the part, the current is interrupted and the pulp strangulated at the foramen in the root, the death resulting being the same as though a ligature had been applied to the part.

If a pulp is not wounded a very small portion of arsenious acid will devitalize it; if the pulp is wounded the quantity required is larger. I have often been surprised to find some cases, weeks after the application, a small part of the pulps sloughed off, the balance remaining alive.

Section 7 was now called, and Dr. Barrett read a paper on "The Origin of Nervous Force." The discussion was limited as the doctor did not want the discussion to assume a metaphysical or subtle phase, but to be confined strictly to the limits of the paper.

FOURTH DAY—MORNING.

SECTION 7 was again called up, but passed after a debate on a resolution to appropriate $200 for a prize essay on etiology.

SECTION 1 presented a report on Reese's metallic base for artificial teeth.

DR. BUCKINGHAM made some remarks on celluloid, as follows:

The best results in the arrangement of the teeth in the plate, can be had from the use of plain teeth and not from sections. It does not hurt the teeth to be ground into any shape, as a better surface can be made by subsequently polishing with pumice-stone and rotten-stone and oil. The color of the teeth can be changed in any way by the use of the mineral paints prepared for the use of pottery-makers. With celluloid for gum, and the means at hand for shaping and coloring the teeth, why can't we obtain better results in making artificial teeth? (The above is the substance of a paper the doctor had prepared but did not read, he merely giving the salient points. He then outlined the method of making celluloid.) To press celluloid into shape, the temperature must be run up to above 290 degrees F. This heat makes the celluloid doughy, it will then easily adapt and will be less likely to shrink after it is removed from the press. The specimens I here show you, have been put in test tubes filled with glycerine and heated to 250, 260, 270, 290, 295 and 300 degrees F., respectively. They show the progress the celluloid makes in softening. At 310 degrees F., it begins to boil and at 320 F., it becomes porous. The working temperature must be between 290 degrees F and 310 degrees F., the medium working temperature being 295 degrees F.

The apparatus used must be capable of giving a uniform temperature for working. This model I show you is for an apparatus with a shell one inch thick, and when heated all through the temperature is uniform. The apparatus can be heated to 320 degrees F., without hurting the piece as the interior of the flask never becomes as hot as the outside. It is for dry heat, not counting the water contained in the plaster. Metallic casts are used to press the plate upon as the plate comes out finished on the inside. The use of celluloid and single teeth offer the greatest variety in the arrangement of the teeth.

Dr. Morgan, If a metallic cast is used and the undercut is great can the plate be warmed and removed without injury to it?

Dr. Buckingham. In such a case it is best to use a skeleton cast, as it can be cut away after pressing.

Dr. Horton. Is the coloring matter in celluloid the same as that in rubber.

Dr. Buckingham. Yes; in celluloid there is but 6-10 of a part in 100, while in rubber there are 36 parts in 100.

Dr. Horton. What ingredient is used to color black rubber?

Dr. Buckingham. Carbon.

Dr. Horton. What is the difference in the transmission of heat in celluloid and rubber?

Dr. Buckingham. I do not know exactly, but I think it is about the same.

Dr. Hurtt. I would say in reply to Dr. Morgan's question about undercuts, that the plate with plain teeth can be sprung off without damage.

Dr. Buckingham. It can be removed by immersing in boiling water; with celluloid, would use plain teeth in all cases.

Dr. Morrison. How does the color of celluloid stand?

Dr. Buckingham. It will not stand in the mouth of a smoker. The celluloid now made is much better than that formerly made, and in most cases will stand for some time.

Dr. Brophy. Has the use of the tin cast any effect in making the color more stable?

Dr. Buckingham. I think it has no specific effect. It makes the surface much harder and gives a better polish.

Dr. Harroun. Is not the hardened surface the very reason that the color is more durable?

Dr. Buckingham. Possibly.

Dr. Atkinson. Whenever we make a substitute for any part of the body, it is necessary that its thermal and other properties be as near those of human tissue as possible. I would like very much to call the attention of the association to Reese's metallic base for artificial teeth. It can be used with plain or gum teeth. Reese has a new method and a new cup for taking impressions. Build wax on the remaining teeth, put in the cup with plaster, allow it to remain till it hardens. The wax comes out with the plaster and then fill the impression with plaster. This gives a correct model of the mouth. The base can be used in many ways, on a platinum plate the teeth can be attached with it and no one can detect the joint between the two metals. The best fit can only be made with a cast metallic base. The Reese metal will also cure the effects arising from the wearing of a rubber plate.

Dr. Robinson. I have here a lining for the palate surface of celluloid and rubber plates. The metal is a mixture of platinum, tin and gold, very little of the latter and considerable of the tin. When the plate is warm the lining mixes with it and cannot be removed, is tough, fibrous and very adaptable. With some modification it also makes an excellent filling material.

Dr. Hurtt. I think that the proceedings this morning have been very disgraceful and degrading. When I came here I expected to hear discussions and transactions by the most eminent men in the profession, for the benefit of the profession, our patients, and most especially for the benefit of the rising generation of dental students. (The Chair called the doctor to order; he appealed to the Association, and continued.) I have always been taught not to engage in any mechanical appliance that would need an advertisement before an Association, in that way begging patronage for it. I am very sorry, indeed, to see Dr. Wm. H. Atkinson, a representative of all that is high and respectable, demean himself by bringing before this Association a patent scheme in which he is financially interested.

If we have a mechanical appliance that we wish to bring before the profession, we must introduce it through some other channel than a direct use of the American Dental Association as an advertising medium. [Loud and continued applause.] Now, in regard to celluloid: I have found that persons using a great deal of alcoholic beverages cannot wear celluloid without losening the plate.

Dr. Buckingham. I disclaim having any interest or private end in view in what I have shown to the Association, and do not want to be classed among those who use the Association to further private schemes.

Dr. Robinson. The world is moved by the omnipotent power of talk. [The Chair called him to order.] I have nothing secret in what I have shown, and have told everything and all about it in all simplicity of purpose. [Again called to order.]

Dr. Buckingham. Dr. Dorrance has an alloy to be used as a solder which he wants to *give* to the profession.

Dr. Dorrance. That is not the question, I have a paper I would like to read and want to know if it is in order.

Voices. Read it.

Dr. Dorrance. I have the alloy and will present it bye and bye. The paper is on the "Ill Effects of Plastic Bases." The doctor read his paper and it was so good and of such a popular nature that the association wished to donate money for its publication and free distribution.

Dr. Allport. I do not want to discuss Dr. Dorrance's paper, nor do I think any one else does. I heartly commend it and want the association to have it printed and distributed to the public in order to educate people and show them the fallacy of cheap mechanical dentistry.

Dr. Dorrance. I feel very much honored by this compliment of the association. Some four or five years ago Dr. Robinson asked me to make some alloy for him. He had made a base for solder, composed of equal parts of silver, copper and zinc. This was excellent, but it needed some improvement as the quantities were not well proportioned. During the past five or six months I have experimented somewhat and have arrived at a more satisfactory result. In making the solder the purest metal only is to be used. The formula is one part of silver, two parts of zinc and three of copper. Melt the copper first, add the silver, and then slowly add the zinc until the fumes have ceased. The proportions in the formula are nearly the combining proportions of the metals as nearly as can be made. The alloy is tough and can be rolled out. It can be used to alloy gold to any carat required. The color of the alloy will follow the color of the gold. For silver solder add one part of the alloy to four parts of silver. To reduce twenty-four carat gold to eighteen add six grains of the alloy. In making a carat solder the alloy will always give the fineness required.

FOUTH DAY—AFTERNOON.

Report of Section 3.

PROF. J. TAFT read a paper on "Dental Literature."

PROF. MAYR. The dental literature as printed at present has a large variety of meanings. It is difficult to make the essays agree with each other. Every author should be particular to state just what he means by the words he uses as terms are more or less technical. A list of such terms is very necessary to explain their exact meaning. Several dental journals published in Spain and Italy are merely repetitions of the English and American journals in the Spanish and Italian languages. Dr. Flagg's "Quiz Questions" is a very funny little book, it will do very nicely to provoke good humor after dinner. It is a conglomeration of empty words. Such books are not adapted for use in the colleges. It belongs to dental literature to say that such and such chemistrys are for dental students. The idea is foolish, chemistry is chemistry and we should beware of such invidious distinctions, for they tend to narrow the mind of the student instead of giving him a true and broad conception of the science of chemistry. Subject passed.

SECTION 2. Dr. Crouse read a paper on "Dental Education."

DR. ROBINSON. I cannot agree with the statement in the paper that we have not made any progress. During the past forty-six years I have witnessed a remarkable progress. The cringing and fawning of many dentists to get a job, is a disgrace that should be rooted out. The dentist in performing his work well confers just as much favor upon his patient as the latter does in giving the dentist employment. The "jewing" down and underbidding that is so much practiced is demoralizing to true dental education. We should all be honest to our patients and to ourselves.

DR. ATKINSON. It is in vain to attempt to elevate the standard of dental education, so long as we follow the old lead. The required one of two, three or ten years' attendance at college has nothing whatever to do with the qualifications of the student. When a dentist cannot diagnose a case, he would do better to shut up or get counsel outside. A man surely knows whether he can diagnose a case or not. It is not until truth and honesty are become integral parts of us that we shall have a true standard of dental education.

Dr. Pierce. Dr. Atkinson has struck the keynote to this question. In my opinion every college should have a board of examiners, independent of the corps of teachers to examine the student and award the diplomas. It is a dangerous precedent to establish to examine a student and give him his degree regardless of time spent in study.

Dr. Babrett. The idea of basing the diploma upon the qualification of the student instead of the time spent at college is very pretty in theory. Until the time arrives when a board of examiners, independent of the teachers, shall grant the diploma we cannot elevate the standard.

Dr. J. Taft. The problems connected with professional education are numerous and difficult and the method of overcoming them is not at all easy. One college pursues one course and another proceeds in some other way. The requirements for entering college should be uniform in all institutions. The power to do this is vested in the profession, but the difficulty is who will take hold of it? Something in this direction can now be done as the profession is far more intelligent than formerly. It is much better to agree on some one thing and carry it out thoroughly than attempt too much and accomplish nothing. There was a proposition for the college faculties to come together and formulate some sort of arrangement to secure this result, but nothing came of it. It has been suggested for some eighteen years past to establish boards of examiners, but the idea lacked support and was abandoned. Such a board is a good thing if the colleges and the profession would only take hold of it and give it their earnest support. It is simply out of the question for one college to establish a board of examiners as students would go to other colleges where the requirements were not so stringent. But if *all* institutions were to adopt this course, the student could not help himself and would be obliged to submit.

Dr. Allport. The idea in Chicago has not been to establish a dental college at all. The object is to establish dental chairs in the faculties of the medical colleges, and it has been done in six out of the seven medical schools in the city. The anatomy and physiology of the teeth together with their diseases, will be taught in order that physicians will know something about the mouth To dentists *per se* these chairs mean nothing, but to physicians are of great importance. Physicians can then better appreciate dental diseases and see the necessity of consulting a dental surgeon for treatment.

Dr. Buckingham. There are necessarily many variations in the teachings of the colleges by reason of their being scattered about here and there. So long as a college exists it must live and if the standard is placed too high, students will not patronize it. The income then fails and it cannot be expected that the professors can always teach for nothing.

Dr. W. H. Morgan. It is a snare and delusion to graduate a student at any time when he is supposed to be competent. It does not hurt them a particle to remain in college for a specified time. I take exceptions to the idea of a board of examiners outside of the corps of teachers, for none know the students so well or are so competent to judge of their qualifications as those who taught them.

Dr. Brophy. What is the reason that a physician does not know anything about dental diseases? Because the college he attended did not embrace any dentistry in its curriculum of studies. Dentistry has a higher aim than the mere filling of teeth and making plates. It aspires to surgery. It is not the intention in Chicago to start a dental college, but to give graduates of medicine some knowledge of the higher dentistry. Dentally educated physicians will do more for the teeth of the future than all the dentists combined.

Dr. Kulp. In Iowa, a medical college taught a little dentistry, and after two sessions it was found that the young M.D.s were practicing as dentists. They had learned some how or other to take impressions and sent the models to the "Iowa Dental Manufacturing Company" to have the plate manufactured as any article of trade might be. The teaching of dentistry in medical colleges will in the future prove a curse.

Dr. Allport. Dr. Kulp is over fearful. The greatest quacks in Chicago are graduates of dental colleges. We cannot help that. Is it anything against the colleges? No, it is the fault of the individual. The idea of building up a profession on a segment of a profession is a humbug. We have been at that sort of thing long enough. Let the foundation be broad and deep. The dental colleges are entitled to great credit, but they lack the breadth of education that a medical college will give.

Adjourned to meet the first Tuesday of August, 1883, at Niagara Falls.

